# Necroptosis in Down Syndrome

**DOI:** 10.1038/s41419-026-09035-y

**Published:** 2026-06-23

**Authors:** Hymavathi Reddy Vari, Domenico Praticò

**Affiliations:** Department of Neural Sciences, Lewis Katz School of Medicine, Temple University, Philadelphia, PA 19140 USA

**Keywords:** Neuroscience, Biochemistry

## Abstract

Necroptosis is a form of controlled cell death implicated in neuronal loss observed in neurodegenerative diseases such as Alzheimer’s disease. Down syndrome (DS) is also characterized by the presence of neuronal cell loss, but the underlying mechanisms remain unclear. Brain tissue from a mouse model of DS, Ts65dn mice, and subjects with DS were assessed for levels of necroptosis markers including receptor-interactive protein kinase 1 and 3, necroptosis executor mixed lineage kinase domain-like protein and long non-coding RNA MEG. While no differences were observed between the Ts65dn and wild type mice at a young age, levels of these markers were significantly elevated in the brains of old DS mice when compared with matched wild type controls. Assessment of post-mortem brains from DS subjects also revealed a significant increase in these necroptosis markers. Our study is the first report showing the presence of necroptosis markers in the brains of a mouse model of DS and in DS subjects. These findings support the novel idea that this form of cell death should be also considered for developing novel therapeutic strategies for DS.

## Introduction

Down syndrome (DS) is the most common form of genetically defined intellectual disability affecting 1 in 700 live birth and deriving from a partial or complete triplication of the human chromosome 21 [1, 2]. DS individuals have a significantly increased risk to develop a neuropathologic phenotype similar to the one found in Alzheimer’s disease (AD) brains, including the presence of abundant amyloid-β peptide deposits, tau neurofibrillary tangles, synaptic dysfunction and pathology, and neuronal loss [3]. Although this increased risk is generally considered secondary to a gene-overdosage effect of the amyloid precursor protein located on chromosome 21, the exact mechanisms involved in the onset and development of these pathologic features are still under investigation [4, 5]. Neuronal cell death and the resulting cellular loss in DS have recently become a focal point of research. Like for AD, post-mortem studies of DS brains consistently demonstrate reductions in both brain volume and neuronal cell count. Emerging evidence suggests that the reduced neuronal cell count observed in adult with DS may also result from progressive cell death [6–8].

Like other cells, long-lived neurons can undergo cell death trough two major mechanisms: apoptosis and necrosis. While apoptosis is a form of programmed cell death, necrosis is an unregulated form of it [9]. In recent years, a new type of programmed cell necrosis called necroptosis was identified. Interestingly, recent work shows that necroptosis can be influenced and activated by a variety of micro-environmental factors [10]. Three key proteins have been identified as directly regulating and orchestrating the onset and completion of this pathway within the cell: receptor-interacting serine/threonine-protein kinases 1 and 3 (RIPK1, PIPK3), and the mixed lineage kinase (MLKL) [11]. This type of cell death is typically initiated by the activation of the necrosome, a multiprotein complex formed by the phosphorylated forms of these three proteins: p-RIPK1, p-RIPK3, and p-MLKL [12].

In recent years, studies have shown the occurrence of necroptosis in amyotrophic lateral sclerosis, multiple sclerosis, and AD [13–16]. However, no evidence is available that necroptosis is present also in DS. To address this question, we used brains from a well-established transgenic mouse model of DS, the Ts65dn mice and post-mortem brain tissues from subjects with DS.

## Results

### Markers of necroptosis in brains from DS mice

**Fig. 1:**
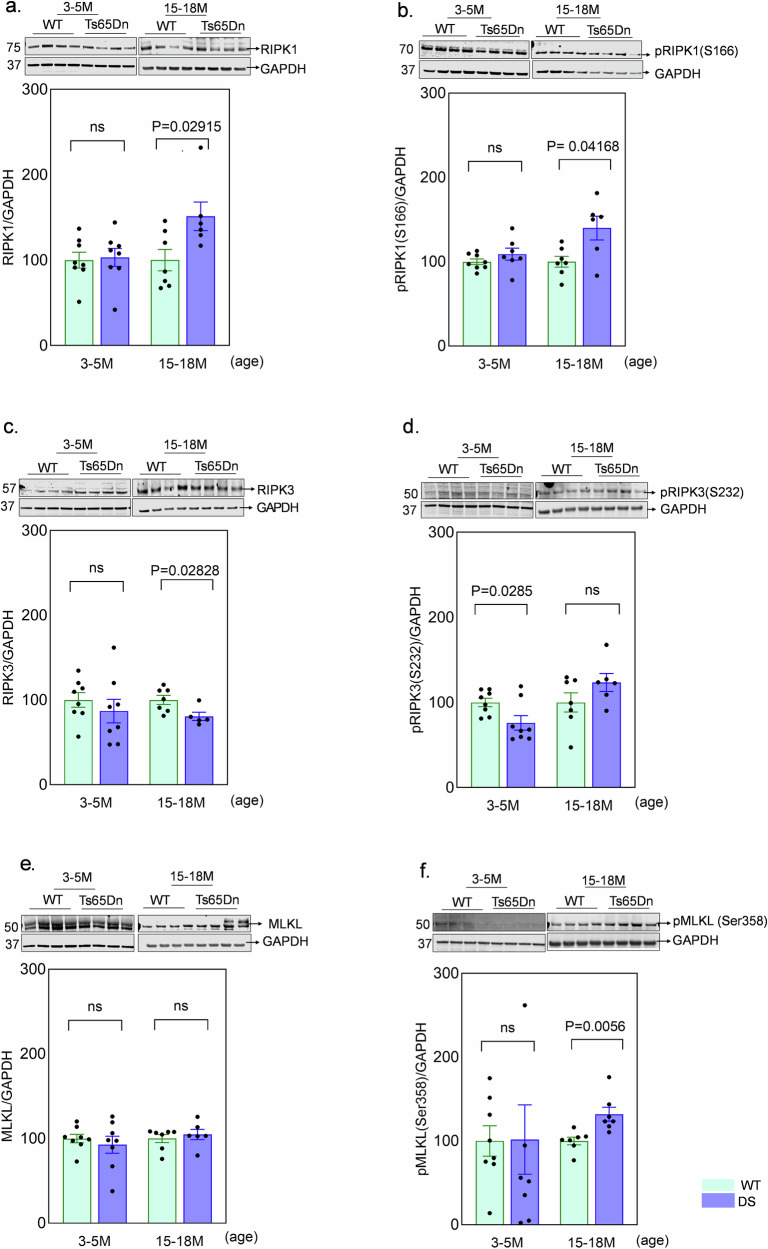
Necroptosis protein markers are increased in DS mice brains. Quantitative analysis of necroptosis-related protein expression in brain cortices of WT and DS, across two age groups: 3–5 M and 15–18 M, expression levels of **a** RIPK1, **b** pRIPK1(S166), **c** RIPK3, **d** pRIPK3(S232), **e** MLKL, **f** pMLKL(S358) with GAPDH used as a loading control. For the 15–18 M age group, the membrane used to detect pMLKL (S358) and pRIPK3 (S232) was stripped once and reprobed for total MLKL and RIPK3 respectively. For the 3–5 M age group, the membrane used for pRIPK3 was similarly stripped once and reprobed for total MLKL. Statistical significance was assessed using unpaired, two-tailed student’s t-test. Data are presented as mean ± SEM (*n* = 6-8 per group); *P* values are indicated where significant (*P* ≤ 0.05).

To assess the presence of biochemical signature of cell necroptosis in DS mice compared with WT controls, we investigated some of the key molecules of this pathway in the brain frontal cortex of these mice at two different age points (3–5 months, young; and 15–18 months, old). As shown in Fig. 1a, we observed that there was no difference in the level of total RIPK1 in the cortex of young WT when compared with young DS mice. By contrast, in the old DS mice these levels were significantly elevated when compared with old WT. While levels of pRIPK1(S166) were not different in the cortex of DS versus WT mice at younger age, a significant difference was observed when the old DS mice were compared with matched WT, suggesting enhanced levels of RIPK1 in DS mice as they age (Fig. 1b).

Total RIPK3 protein levels were comparable between young DS and WT mice, indicating no early changes in RIPK3 expression. However, aged DS mice exhibited a significant reduction in RIPK3 levels compared to aged WT controls (Fig. 1c). Phosphorylated RIPK3 at S232 (pRIPK3) was reduced in young DS mice relative to age-matched WT mice. No significant differences in pRIPK3(S232) levels were observed between aged DS and WT mice (Fig. 1d).

To assess the terminal effector of necroptosis, we examined total MLKL and its phosphorylated form (pMLKL at Ser358) in the frontal cortical tissues of young and aged DS and WT mice. As shown in (Fig. 1e), total MLKL levels did not differ significantly between DS and WT mice in either age group. By contrast, while analysis of phosphorylated MLKL revealed no significant differences in young DS mice compared to WT controls, aged DS mice exhibited a marked increase in pMLKL (Ser358) levels relative to aged WT mice (Fig. 1f).

**Fig. 2:**
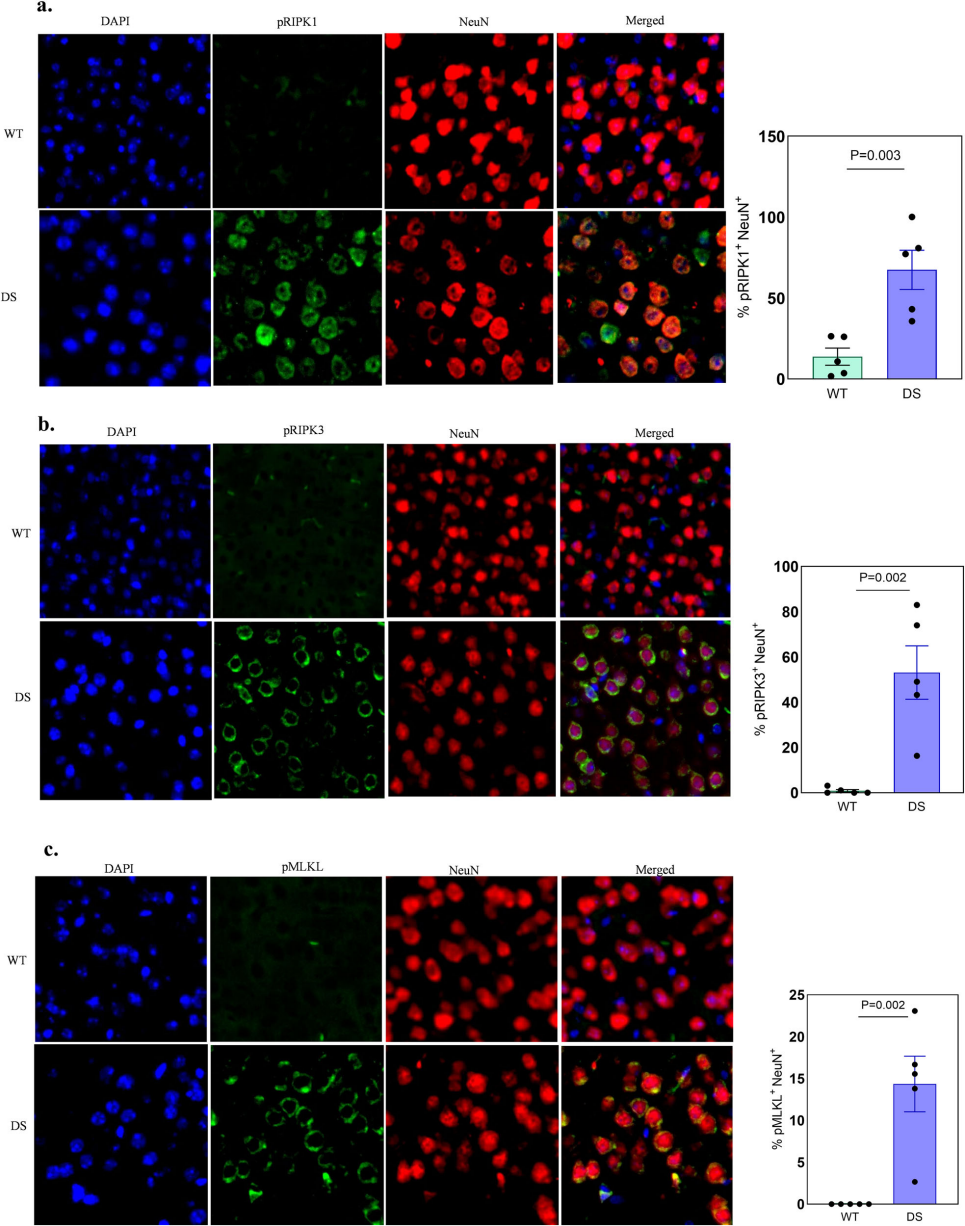
Necroptosis markers are increased in neuronal cells of DS mice. Representative immunofluorescence microscopy images for phosphorylated necroptosis markers and NeuN⁺ neurons in 12-month-old WT (*n* = 5) and DS (*n* = 5) mice. Panels **a–c** show single immunoreactivity to each protein (pRIPK1, pRIPK3, and pMLKL), and then NeuN colocalization with each of them (merge). For each animal, three sections were analyzed and averaged. Group comparisons were performed using unpaired, two-tailed student’s t-test. Data are presented as mean ± SEM (*n* = 5 per group), with P ≤ 0.05 considered significant. DAPI (blue) marks nuclei, NeuN (red), and necroptosis markers (green).

We also measured the levels of pRIPK1, pRIPK3 and pMLKL by immunofluorescence in the old mice group. As shown in Fig. 2, we found that compared with WT controls, pRIPK1, pRIPK3 and pMLKL immunoreactivities were higher in brains sections of DS mice (Fig. 2a–c). Interestingly, the increased signals for these markers of necroptosis were mainly observed in neuronal cells as demonstrated by the higher degree of co-localization between them and the NeuN positive cells.

**Fig. 3:**
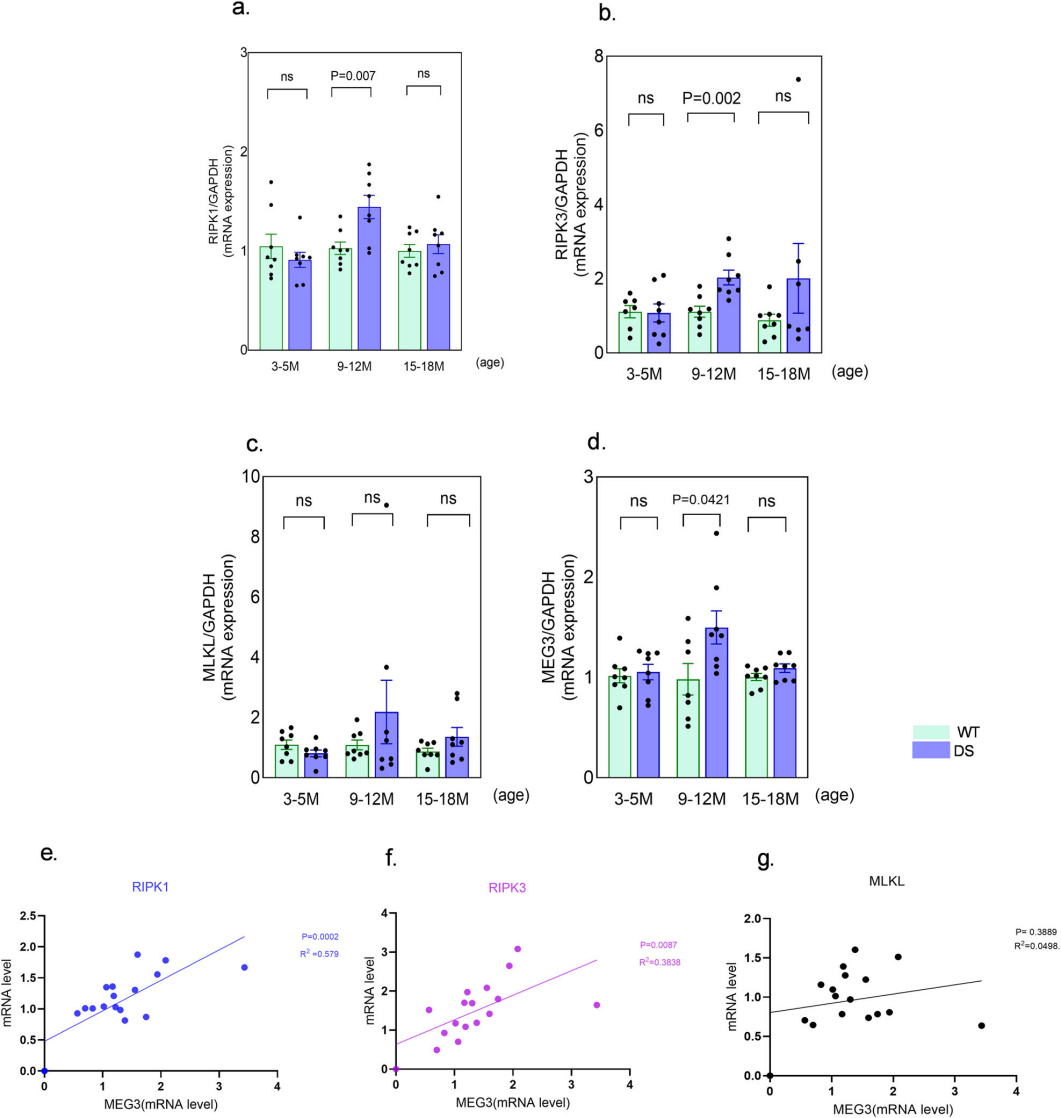
RNA messengers of necroptosis markers and MEG3 in DS mice brains. mRNA expression analysis of necroptosis-related genes and MEG3 in brain cortices of WT and DS mice across three age groups: 3–5 months, 9–12 months, and 15–18 months. Expression levels of **a** RIPK1, **b** RIPK3, **c** MLKL, and **d** MEG3 were normalized to GAPDH. Statistical significance was assessed using unpaired, two-tailed student’s t-test. Data are presented as mean ± SEM (*n* = 8 per group); *P* values are indicated where significant (*P* ≤ 0.05). Correlation analysis between MEG3 and necroptosis markers in the 9–12-month-old group are shown in **e** RIPK1, **f** RIPK3, and **g** MLKL across all groups. The data were tested for normality and analyzed using the Pearson correlation method.

Next, we examined mRNA expression levels of these 3 proteins in the same brain regions of these mice across three age groups (3–5, 9–12, and 15–18 months) using semiquantitative RT-PCR. No significant differences were observed in RIPK1 and RIPK3 expression at younger and older ages; however, both were significantly upregulated in 9–12-month-old DS mice, while MLKL expression remained unchanged across all age groups (Fig. 3a–c).

Next, we assessed the levels of MEG3, a long noncoding RNA which previously was reported to be an activator of necroptosis [17]. Our RT-PCR analysis showed that its levels were significantly upregulated in 9–12-month-old DS mice, the same age for the increase of the mRNAs for the necroptotic markers (Fig. 3d). To further explore the potential regulatory relationship between MEG3 and necroptosis-related genes in DS, we performed correlation analyses between MEG3 expression and the mRNA levels of RIPK1, RIPK3, and MLKL in the 9–12-month-old age group. MEG3 expression was significantly correlated with both RIPK1 and RIPK3 (Fig. 3e, f). By contrast, no significant correlation was observed between MEG3 and MLKL expression (Fig. 3g).

### Cell loss in DS mice

**Fig. 4:**
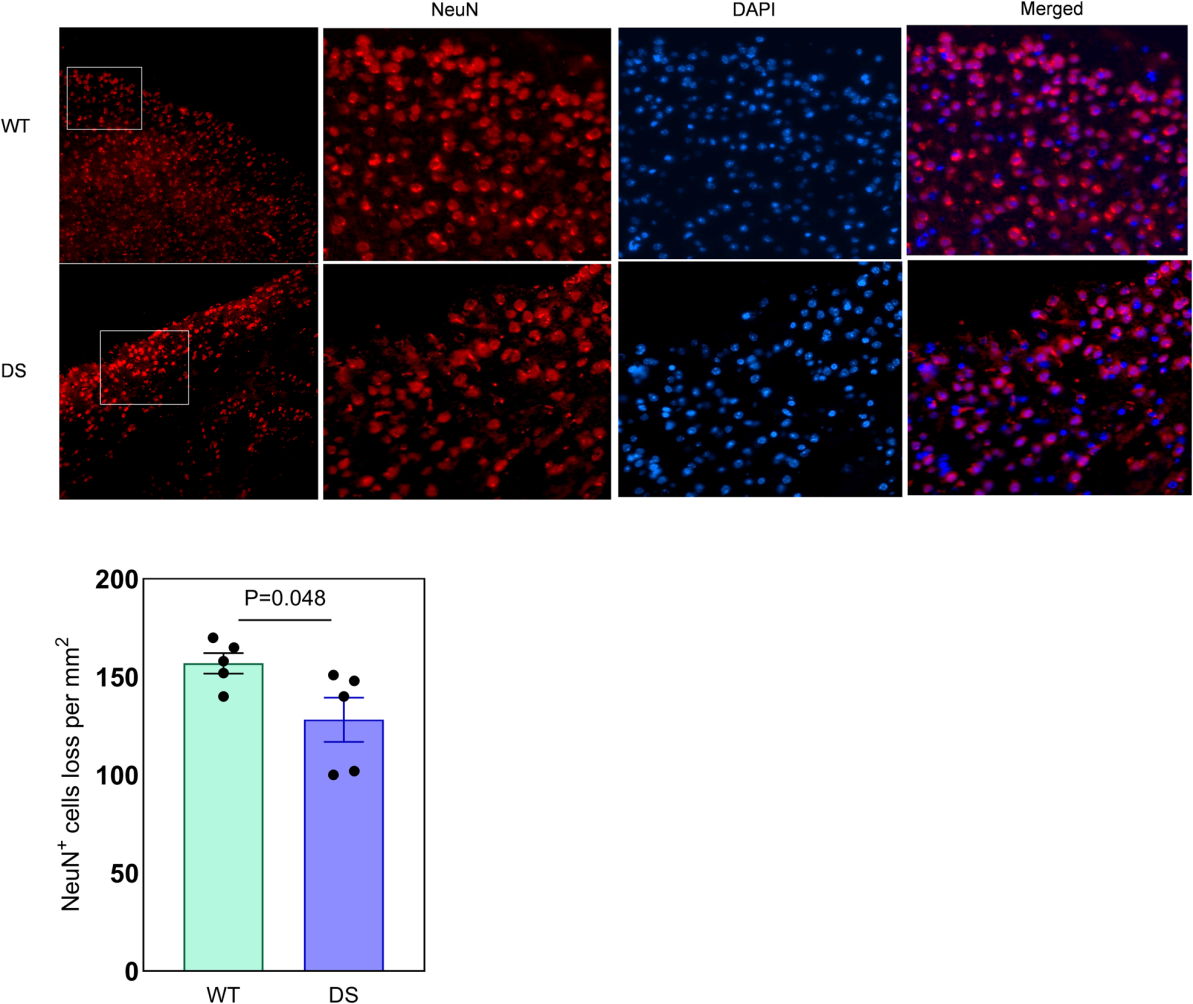
Decrease in neuronal density in DS mice brains. Representative fluorescence microscopy images and quantification of NeuN-positive neuronal cells in WT and DS mouse brain tissue. The top panels display NeuN (red), DAPI (blue), and merged channels for WT and DS samples, illustrating neuronal nuclei and overall cell density. White boxes mark equivalent regions of interest (ROIs) selected for quantitative analysis across samples. NeuN-positive neurons were quantified using ImageJ/Fiji following a standardized workflow to ensure consistency across WT and DS groups. For each image, the NeuN channel was first isolated and converted to an 8-bit grayscale image. Background signal was reduced using the Subtract Background function, and a consistent threshold was applied (Otsu or manually defined based on control images) to segment NeuN-positive nuclei. The thresholded image was then binarized, and the “Analyze Particles” tool was used to automatically count NeuN-positive cells within each ROI. DAPI staining was used to confirm nuclear localization and ensure accurate segmentation. Quantified NeuN+ cell counts from each ROI were averaged for each animal, for each animal, three slides were analyzed and the average value was used, and group means were calculated (*n* = 5 per group). Data are presented as mean ± SEM. Statistical comparison using an unpaired, two-tailed student’s t-test revealed a significant reduction in NeuN-positive neurons in DS samples compared to WT (*P* = 0.048).

Immunofluorescence analysis of brain cortices revealed a reduction in neuronal density in DS mice compared to WT controls. Quantification of NeuN-positive cells demonstrated a significant decrease in the number of neurons in DS mice across the examined regions, as shown in Fig. 4.

### Markers of necroptosis in brains from DS Subjects

**Fig. 5:**
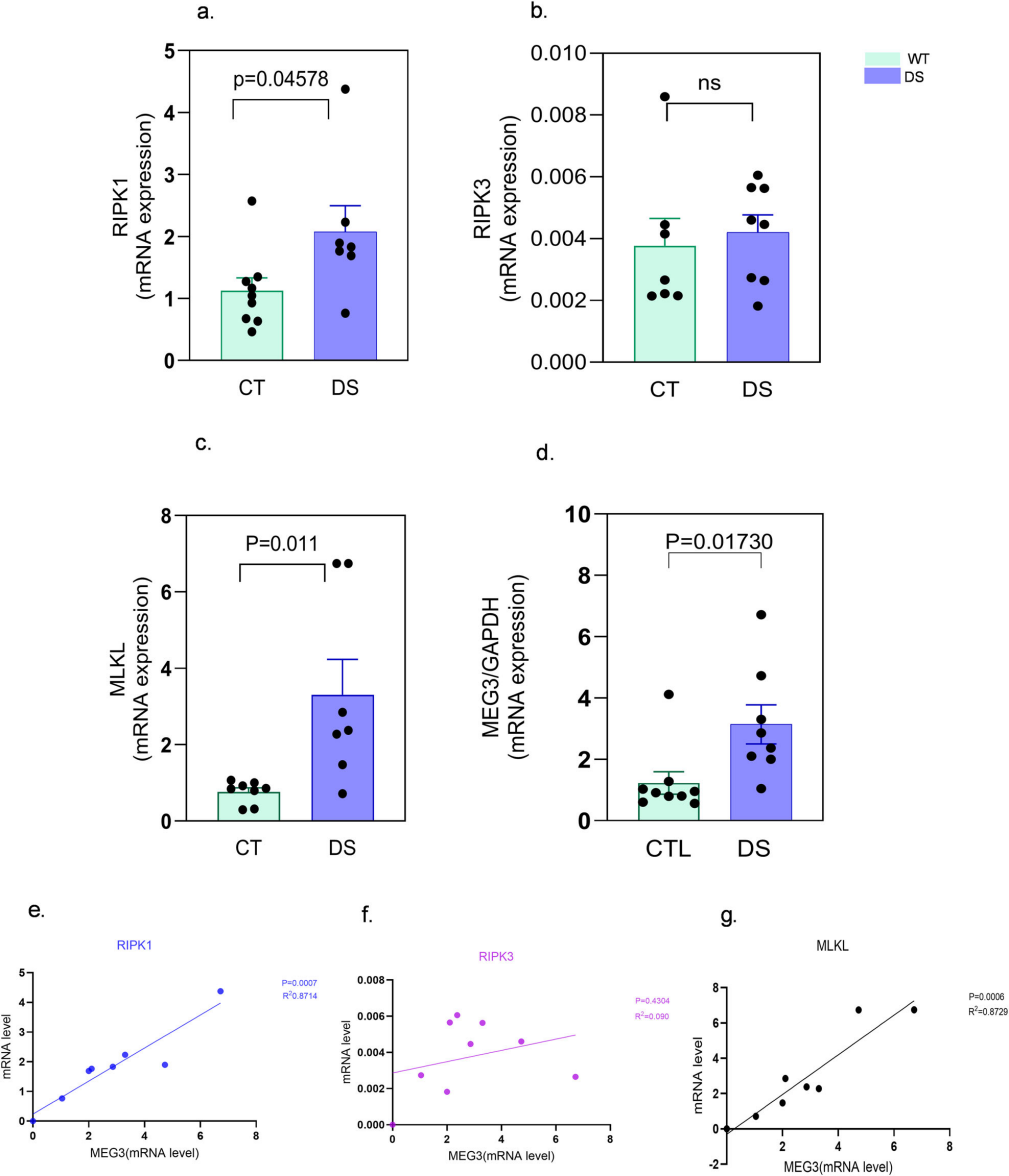
Increased expression of necroptosis-related genes in human brain cortices from subjects with DS. Quantitative analysis of mRNA expression levels for necroptosis markers **a** RIPK1, **b** RIPK3, **c** MLKL and MEG3 **d** were performed in cortical tissue from CT and DS individuals. Expression levels were normalized to GAPDH. Data are presented as mean ± SEM with *n* = 9 for CT and *n* = 8 for DS group. Statistical significance was determined using unpaired, two-tailed Student’s t-test, with *P* values indicated where significant (*P* ≤ 0.05). Based on ROUT outlier detection in GraphPad Prism, one DS sample was excluded from RIPK1 mRNA (panel **a**), two control samples were excluded from RIPK3 mRNA (panel **b**), and one control and one DS sample were excluded from MLKL mRNA (panel **c**). Correlation analyses between MEG3 and necroptosis-related genes in DS samples are shown for **e** RIPK1, **f** RIPK3, and **g** MLKL. The data were tested for normality and analyzed using the Pearson correlation method.

Human frontal cortical tissue samples were assessed for the expression of necroptosis-related markers genes and the non-coding RNA MEG3. Compared with controls, DS brains had a significant increase in RIPK1 and MLKL mRNA levels (Fig. 5a, c), while RIPK3 expression remained unchanged between the two groups (Fig. 5b). Additionally, like with the DS mice brains, we detected a marked upregulation of MEG3 in the brains from DS subjects when compared with matched controls (Fig. 5d). To further investigate the relationship between MEG3 and necroptosis-related genes, we performed correlation analyses between MEG3 expression and mRNA levels of RIPK1, RIPK3, and MLKL. As shown in Fig. 5e and g, MEG3 expression exhibited a significant positive correlation with both RIPK1 and MLKL. In contrast, no significant correlation was observed between MEG3 and RIPK3 expression (Fig. 5f).

## Discussion

DS is characterized by intellectual disability and early-onset neurodegeneration, particularly affecting the hippocampus and cortex [8, 18]. Among the various aspects of the DS neuropathologic phenotype, in recent years neuronal cells loss has attracted a lot of attention. Studies in post-mortem brains from DS subjects have clearly and consistently indicated a reduction in cell number [8, 19]. Despite this evidence, the exact mechanism responsible for it is still largely unclear. In fact, while some aspects could be secondary to a developmental deficit and reduced neurogenesis, emerging evidence indicates that in DS the lower cell count could also be secondary to cell death later in life [20]. The identification of the pathways leading to neuronal loss in DS is of fundamental importance for two reasons: first, to establish what mechanism is responsible for it; second, to develop novel therapeutics targeting it that could slow or block some of the neurodegenerative processes of the syndrome.

Like other cells, neurons can undergo cell death trough two major mechanisms: apoptosis and necrosis. Apoptosis is a form of programmed cell death, on the other hand necrosis is an unregulated form of it [21, 22]. In recent years, a new type of programmed cell death called necroptosis has been identified. Necroptosis is a regulated form of necrotic cell death mediated by the coordinate work of 3 major molecular players (RIPK1, RIPK3, and MLKL), which recently has emerged as a contributor to several neurodegenerative diseases [23]. Unlike apoptosis, necroptosis is caspase-independent and often associated with inflammation, making it a potential driver of neuronal death in DS [24]. In our study we aimed to evaluate necroptosis markers in the brain frontal cortices of Ts65Dn mice, a transgenic model of DS, at two distinct age points: young and old. To reach this goal, we started our investigation by looking at the protein levels of the 3 major markers of necroptosis: RIPK1, RIPK3, and MLKL.

RIPK1, a key regulator of necroptosis, has been reported increased in neurodegenerative diseases such as AD, Parkinson’s disease, and frontotemporal dementia [25, 26]. In our study, we found that while there were no significant changes for this marker at a young age, its levels, both for the total and phosphorylated isoform, were significantly elevated in the brain frontal cortices of old DS mice when compared with matched old WT mice. These findings support the hypothesis that RIPK1 and pRIPK1(S166) could be involved in the molecular mechanisms driving cell loss and neurodegeneration in the DS brain.

RIPK3 is another molecule involved in necroptosis. We observed that the total level of RIPK3 was lower in the cortex of old but not young DS mice relative to control animals, which may reflect a compensatory downregulation of this protein in response to chronic neuroinflammation or cellular stress. Although we observed a trend towards an increase for the levels of pRIPK3(S232) in aged DS mice, the value did not reach statistical significance. Because of the reduced levels for the total protein in this age group, we speculate that in the aging DS mouse brain there could be an altered signaling balance or a compensatory mechanism responsible for it.

MLKL is considered the terminal effector of necroptosis whose phosphorylation leads to its oligomerization and translocation to the plasma membrane, where it disrupts membrane integrity and releases damage-associated molecular patterns. Previous studies have shown that MLKL deficiency could protect against neurodegeneration in mouse models [27, 28]. In our study, while we did not observe any change for total MLKL we found a significant elevation of pMLKL (Ser358) in the aged DS mice, further supporting that in this model another marker of necroptosis increases with age. The increased pMLKL (Ser358) in aged DS mice suggests that post-translational regulation, rather than transcriptional changes, underlies the changes we observed in the old mice brains. In summary, our data demonstrates a significant increase in this necroptotic marker in aged DS mice. This finding aligns well with previous studies showing that pMLKL (Ser358), rather than total MLKL, increases with age and contributes to neuroinflammation and cell death [29].

To assess whether the necroptotic markers are associated with the neurons in the DS cortex, we performed immunofluorescence analyses. Compared with matched controls, we observed an increase in pRIPK1, pRIPK3, and pMLKL immunoreactivity that largely co-localized with the neuronal marker NeuN in DS mice. These findings indicate that necroptotic signaling components are predominantly localized to neurons in the DS cortex. Our results are in line with similar ones in AD, where necroptosis-related proteins have been detected in neurons from postmortem human AD brains and mouse models [30]. Taking together these observations place our findings within a broader neurodegenerative framework and suggest that necroptotic signaling in the DS brain is also at least in part neuron-associated.

To further validate the observed protein level changes, we examined the mRNA expression of the same key necroptosis markers in the frontal cortices of Ts65Dn mice. Initially, we used brain tissues from the same two age groups (3–5 and 15–18 months) to assess the mRNA levels for RIPK1, RIPK3 and MLKL. Since we did not find any significant difference between WT and DS mice for all 3 mRNAs, we decided to look at an age between those two, namely 9–12 months. Interestingly, we found that at this age levels of mRNA for RIPK1 and RIPK3 were significantly higher in DS brains when compared with WT brains. Although MLKL showed a trend to increase in DS brains, the difference with WT was not significant.

We speculate that the mid-life increases in the necroptotic markers mRNA in DS mice, which precedes the increase in the proteins, may be secondary to the onset of neuroinflammatory processes and early cognitive decline, which are well-documented at this age in DS models [31]. Additionally, the transient nature of RIPK1 and RIPK3 mRNA elevation in DS mice may reflect a critical transition phase in disease progression, where necroptosis signaling is temporarily amplified before stabilizing or being suppressed due to cellular exhaustion or feedback inhibition. Overall, our findings highlight the importance of timing and regulation in necroptosis signaling during the development of the DS-related neuro-pathological phenotype in these mice.

To confirm that indeed the mouse model of DS we implemented in our study manifests neuronal loss, we used a semiquantitative approach by using immunofluorescence microscopy. NeuN is a well-established marker of mature post-mitotic neurons and widely used to assess neuronal loss due to various pathological conditions [32, 33]. The results of this measurement indicated that there is a significant reduction of neurons in the brains of DS mice, which aligns with the observed presence of necroptotic markers in the same animals.

Considering our immunofluorescence studies showing that indeed the signal for the necroptosis markers are elevated in the brains for DS mice and expressed within neuronal cells we speculate that like in AD also in an animal model of DS necroptosis could be a contributor to neuronal loss. However, further studies are necessary to establish the direct and functional role of this programmed type of cell death in the neuronal loss observed in DS.

So far, we have provided biochemical and molecular evidence of the presence of necroptosis markers in the brains of a mouse model of DS. Although most of the available mouse models of a disease reproduce well some of the major features of it, caution should always be taken before translating any findings into the human condition. For this reason, having observed these changes in the brains of a mouse model of DS, next we wanted to see whether the same pattern could also be found in the brains of DS subjects. Indeed, compared with normal controls, frontal cortices from DS subjects had a significant increase in the mRNA levels for RIPK1 and MLKL. Although we observed a trend towards increase for RIPK3 mRNA levels, the difference with controls did not reach statistical significance.

It is important to notice that in the human samples, only mRNA levels were assessed, whereas protein and phospho-protein data for the same markers were not available. For this reason, by contrast with the animal findings, the human data would support only a transcriptional rather than biochemical association with necroptosis.

MEG3 is a long non-coding RNA that recently has emerged as a key regulator of programmed cell death, particularly necroptosis. Recent studies in AD models showed that MEG3 is upregulated in human neurons exposed to amyloid pathology and is sufficient to induce necroptosis by modulating the expression and activity of RIPK1, RIPK3, and MLKL [34]. In our study, we found that in both brain frontal cortices from DS mice as well as DS subjects, the levels of MEG3 were significantly elevated when compared with matched controls. Interestingly, we also observed a direct correlation between the increase in MEG3 levels and the necroptosis markers in the frontal cortices from both DS and DS subjects. Although like any correlation, our analysis shows mainly an association and not a cause effect between the variables considered, it supports the hypothesis that MEG3 could be involved in necroptosis by acting as a transcriptional regulator of RIPK1/RIPK3/MLKL [34].

Previously a study showed that downregulation of MEG3 or pharmacological inhibition of RIPK1/RIPK3/MLKL can rescue neuronal cell loss in xenografted human neurons [34]. Based on this model, MEG3 could serve as a molecular switch that sensitizes neurons to necroptotic death during a critical window of vulnerability in DS. Interestingly, the lack of MEG3 upregulation in aged DS mice (15–18 months), despite ongoing neurodegeneration, suggests that MEG3-mediated necroptosis may be transiently activated during mid-life.

In conclusion, our work is the first direct experimental evidence that necroptosis is present both in brains from a mouse model of DS and subjects with DS. While further studies are necessary to prove a direct involvement of MEG3 in the modulation of RIPK1/RIPK3/MLKL levels, and to identify the potential molecules directly involved in the activation signal(s) of this form of cell death in DS, our work by providing a strong rationale should be used as a work frame towards the discovery and development of therapies against necroptosis in DS.

## Materials and methods

### Animals

Animal care and procedures were approved by the Institutional Animal Care and Usage Committee. Ts65Dn mice (stock 005252) and wild type mice (WT) were purchased from Jackson laboratories. Both male and female mice were used. For each age group, WT and DS cohorts included *n* = 4 males and *n* = 4 females. Mice were always kept in a pathogen-free environment, on a 12-h light/dark cycle and fed chow and water ad libitum.

### Tissue harvest

As previously described [35, 36] all mice were euthanized and brains harvested after intraventricular perfusion with ice-cold PBS buffer, ethylenediaminetetraacetic acid (EDTA) and phosphatase inhibitor cocktail. The frontal cortex was dissected, and immediately stored at −80 °C.

### Postmortem human brain samples

Postmortem frontal cortex tissues from DS and matched control (CT) subjects were obtained from the University of Maryland Brain and Tissue Bank. Patient information is listed in Table 1.

**Table 1:** Demographic information on the brain samples from subjects with DS and controls (CT) subjects used in our study.

Sample ID	Sex	Age	Group	PMI	Cause of Death
5926	M	21	CT	27	Cardiac Arrythmia
5342	M	22	CT	14	Multiple Injuries
5958	M	22	CT	24	Dilated Cardiomegaly
6061	M	24	CT	33	Acute and Chronic Lung Disease
5288	M	27	CT	13	Congestive Heart Failure
5753	M	28	CT	28	Dilated Cardiomegaly
6096	M	28	CT	7	Multiple Injuries
4590	M	20	CT	13	No clinical brain diagnosis found
5563	M	29	CT	22	Pulmonary Thromboembolism
2854	M	15	DS	14	Complications of the Disorder
1960	M	19	DS	14	Complications of the Disorder
5277	M	19	DS	26	Diabetic Ketoacidosis
707	M	22	DS	15	Pneumonia
753	M	23	DS	24	Cardiac Arrest
5341	M	25	DS	24	Unknown
5713	M	25	DS	22	Pneumonia
6135	M	55	DS	12	Complications of the Disorder

*PMI* Postmortem Interval, *M* Male, *CT* Control, *DS* Down syndrome

### Immunoblotting

Proteins were extracted from the samples using RIPA buffer (Thermo Fisher Scientific, Cat. No. 89900) supplemented with phosphatase inhibitor cocktail (Thermo Fisher Scientific, Cat. No. 78420), sonicated and centrifuged at 45,000 rpm for 45 min at 4 °C, and supernatants used for immunoblotting analysis. Protein concentration was determined using the BCA Assay Kit (Invitrogen, Cat. No. 23225), following the manufacturer’s instructions. Protein samples were denatured by adding dithiothreitol (DTT) (Bio-Rad, Cat. No. 1610611) and 4x loading dye (Bio-Rad, Cat. No. 1610747), followed by heating at 95 °C for 5 min. Equal amounts of protein were loaded onto a 10% Bis-Tris Criterion^TM^ XT precast gel (Bio-Rad, Cat. No. 3450113 or 3450112) and electrophoresed for 1 h at 170 V. Following electrophoresis, proteins were transferred to a Nitrocellulose membrane (Thermo Fisher Scientific, Cat. No. 88518) using a semi-dry transfer system for 1 h at 80 V. The membrane was then blocked with LI-COR Odyssey Blocking Buffer (LI-COR, Cat. No. 927–40000) for 1 h at room temperature to prevent non-specific binding. After blocking, the membrane was incubated overnight at 4 °C with the following primary antibodies: pRIPK1 (S166) (1:400; Cell Signaling, Cat. No. 31122S), RIPK1 (1:400; ProSci, Cat. No.5389), pRIPK3 (S232) (1:400; Thermo Fisher, Cat. No. PA5-105701), RIPK3 (1:400; Thermo Fisher, Cat. No. PA-519956), pMLKL (Ser358) (1:400; Thermo Fisher, Cat. PA5-105678), MLKL (1:400; Proteintech, Cat. No. 66675-1-Ig), and GAPDH (1:1000; Cell Signaling Technology, Cat. No. 5174). Following overnight incubation at 4 °C, the membrane was washed three times with TBST (Tris-buffered saline with 0.1% Tween 20) to remove unbound antibodies. The membrane was then incubated with IRDye-conjugated secondary antibody (LI-COR, Cat. No. 926-32212 or 926-32211, depending on host species) at 1:10000 dilution for 1 h at room temperature, followed by three washes with TBST. Finally, the protein bands were visualized using an infrared imaging system (LI-COR Odyssey), and the images were analyzed and quantified using Image studio lite version 5.2.

In some cases, when multiple proteins were probed on the same membrane, blots were stripped using 1× New Blot Nitrocellulose Stripping Buffer (LI-COR, Cat. No. 928-40030) for 5 min at room temperature, followed by three washes with Milli-Q water. Membranes were re-blocked and sequentially re-probed with primary antibodies as described above. Each antibody incubation was followed by TBST washes and detection*.*

### RT-PCR

Total RNA was isolated from mice frontal cortex, as well as from human frontal cortex tissue by using the miRNeasy Mini Kit (Qiagen, Cat. No. 217004), following the manufacturer’s protocol. Approximately 250 ng of total RNA from each sample was reverse transcribed using the High-Capacity cDNA Reverse Transcription Kit (Thermo Fisher Scientific, Cat. No. 4368814). The resulting cDNA was used for qPCR with TaqMan™ Gene Expression Assays for RIPK1 (Assay ID: Mm00436354_m1), RIPK3 (Assay ID: Mm00444947_m1), MLKL (Assay ID: Mm01244222_m1), MEG3 (Assay ID:00522599_m1) and GAPDH (Assay ID: Mm99999915_g1), For Human gene expression assay for RIPK1 (Assay ID: Hs01041869_m1), RIPK3 (Assay ID: Hs00179132_m1), MLKL (Assay ID: Hs04188505_m1), MEG3 (Assay ID: Hs00292028_m1) and GAPDH (Assay ID: Hs99999905_m1), using the TaqMan™ Fast Advanced Master Mix (Applied Biosystems, Cat. No. 4444557). PCR amplification was carried out under the following conditions: 50 °C for 2 min, 95 °C for 20 s, followed by 40 cycles of 95 °C for 3 s and 60 °C for 30 s. Gene expression was quantified using the ΔΔCt method with normalization to GAPDH.

### Immunofluorescence

Brains from 12-month-old WT (*n* = 5) and DS (*n* = 5) mice were perfused with PBS and fixed overnight in 4% paraformaldehyde (Thermo Scientific, Cat. No. J61899). Tissues were dehydrated using a KEDEE processor, embedded in paraffin, cooled for 4 h, and sectioned at 8 µm. Sections were baked at 37 °C overnight. Deparaffinization was performed with xylene (2×5 min), followed by rehydration through graded ethanol (100% X2, 95%, 70%, 50%; 2-3 min each). Antigen retrieval was carried out in 10 mM citrate buffer (p^H^-6) at boiling temperature for 40 min, then cooled to room temperature. Sections were permeabilized with 0.1% Triton X-100 in PBS for 10 min and blocked with 5% BSA containing 0.1% Triton X-100 for 1 h at room temperature. For Necroptosis markers colocalization with NeuN used following primary antibodies: pMLKL (1:200; cell signaling Cat. No. 37333), pRIPK1 (1:100; Thermofisher, Cat. No. PA5-104645), pRIPK3 (1:200; Cell signaling, Cat. No. 91702), NeuN (1:200; Abcam Cat. No. 104224) and used following secondary antibodies: Alexa Fluor 488-conjugated goat anti-rabbit IgG (1:500, Abcam, cat. No. 150061) and Alexa Fluor 647-conjugated goat anti-mouse IgG (1:500, Abcam, cat. No. 150113). NeuN primary antibody (1:500; Cell Signaling, Cat. No. 24307) was applied overnight at 4 °C. After washing with PBST (3×5 min), Alexa Fluor® 647-conjugated secondary antibody (1:1000; Abcam, Cat. No. 150083), was added for 1 h in the dark. Slides were washed again (3×5 min), counterstained with DAPI (1:1000; Invitrogen, Cat. No. D1306), and mounted using ProLong™ glass antifade mountant (Invitrogen, Cat. No. P36980). Images were acquired using a Nikon fluorescence microscope at 20X magnification and analyzed with ImageJ/Fiji following a standardized workflow to ensure consistency across WT and DS groups. For each image, each channel was first isolated and converted to an 8-bit grayscale image. Background signal was reduced using the Subtract Background function, and a consistent threshold was applied. For each quantification, three matched coronal sections per animal were analyzed by an observer blinded to group allocation.

### Statistical analysis

All graphs were created using GraphPad Prism version 10, which was utilized for data visualization and statistical analysis. The data were tested for normality. Results were presented as mean ± SEM. Significance was set at *p* < 0.05. Unpaired, two-tailed student’s t-test was applied when variable means were compared between two groups. The 3–5-month and 15–18-month mouse groups were independent cohorts, and no animal was included in more than one age group. Because the study design does not form a crossed genotype x age structure, a two-way ANOVA cannot be applied. Within each age group, the planned comparison was between WT and DS mice only, therefore, unpaired, two-tailed student’s t-test were used to compare these independent groups. No statistical analyses were performed across age groups, and age was not treated as a factor in any model. Outlier values in the human mRNA datasets (RIPK1, RIPK3, and MLKL) were identified using GraphPad Prism’s ROUT method and excluded before statistical analysis. The final sample numbers reported in Fig. 5 reflect these exclusions.

## Supplementary information

Original Data

## Data Availability

Data and materials will be available upon reasonable request.
