# Supplementary material for: Necroptosis in Down Syndrome
Source: Cell Death Dis. 2026 Jun 23;17(1):598. doi: 10.1038/s41419-026-09035-y (PMC13309616; doi:10.1038/s41419-026-09035-y)
Supplement: Supplementary file 1 — Original Data [file 41419_2026_9035_MOESM1_ESM.pdf]

Figure-1(a)

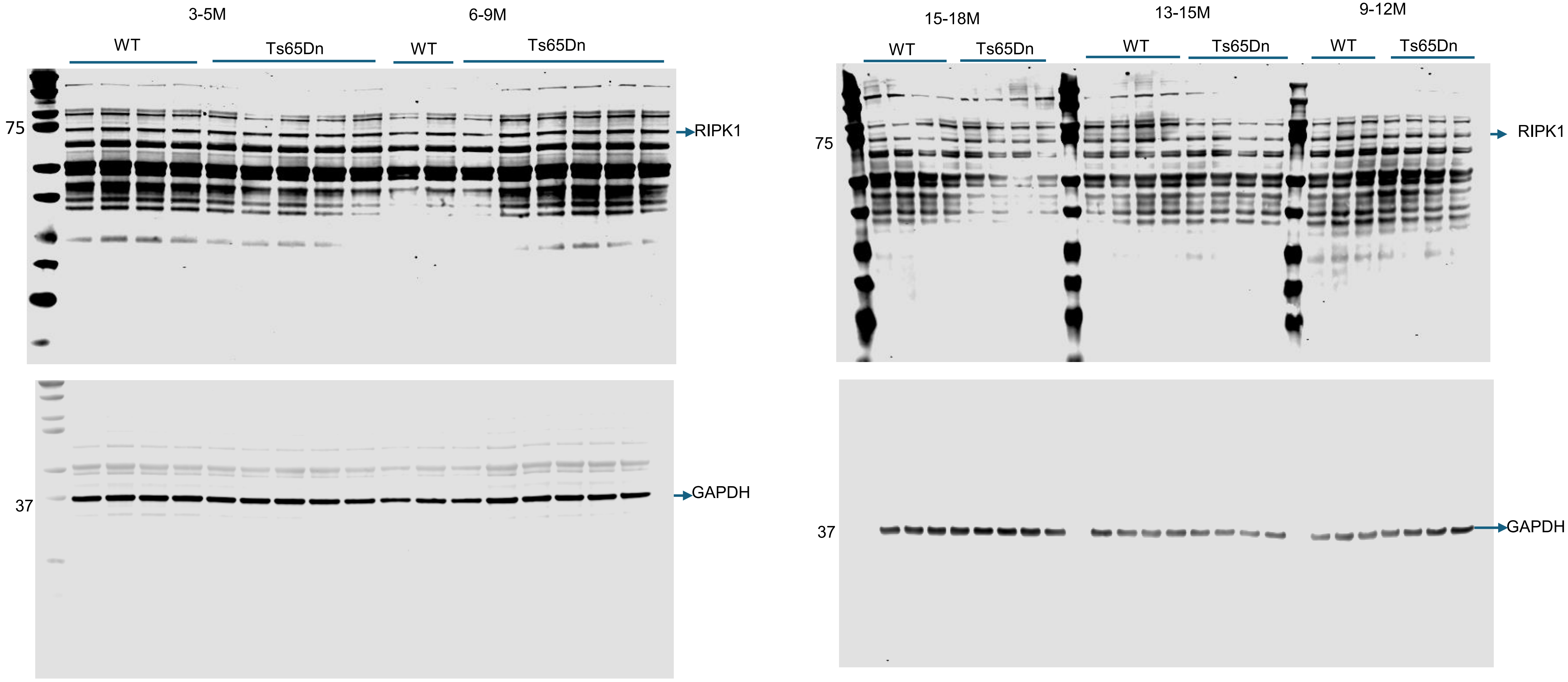
3-5M
6-9M
WT
Ts65Dn
WT
Ts65Dn
75
RIPK1
37
GAPDH
15-18M
13-15M
9-12M
WT
Ts65Dn
WT
Ts65Dn
WT
Ts65Dn
75
RIPK1
37
GAPDH

Figure-1(b)

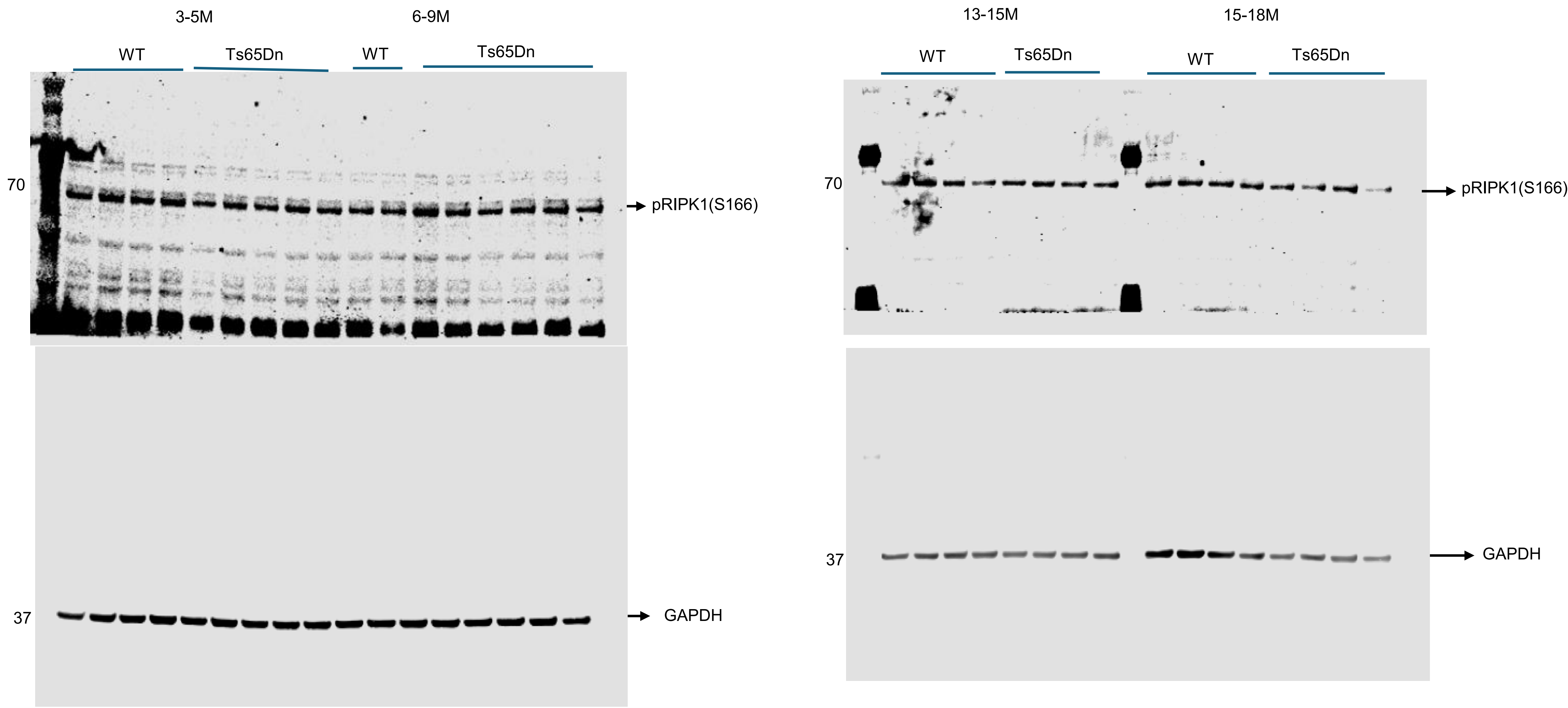
3-5M
6-9M
WT
Ts65Dn
WT
Ts65Dn
70
pRIPK1(S166)
37
GAPDH
13-15M
15-18M
WT
Ts65Dn
WT
Ts65Dn
70
pRIPK1(S166)
37
GAPDH

Figure-1(d)

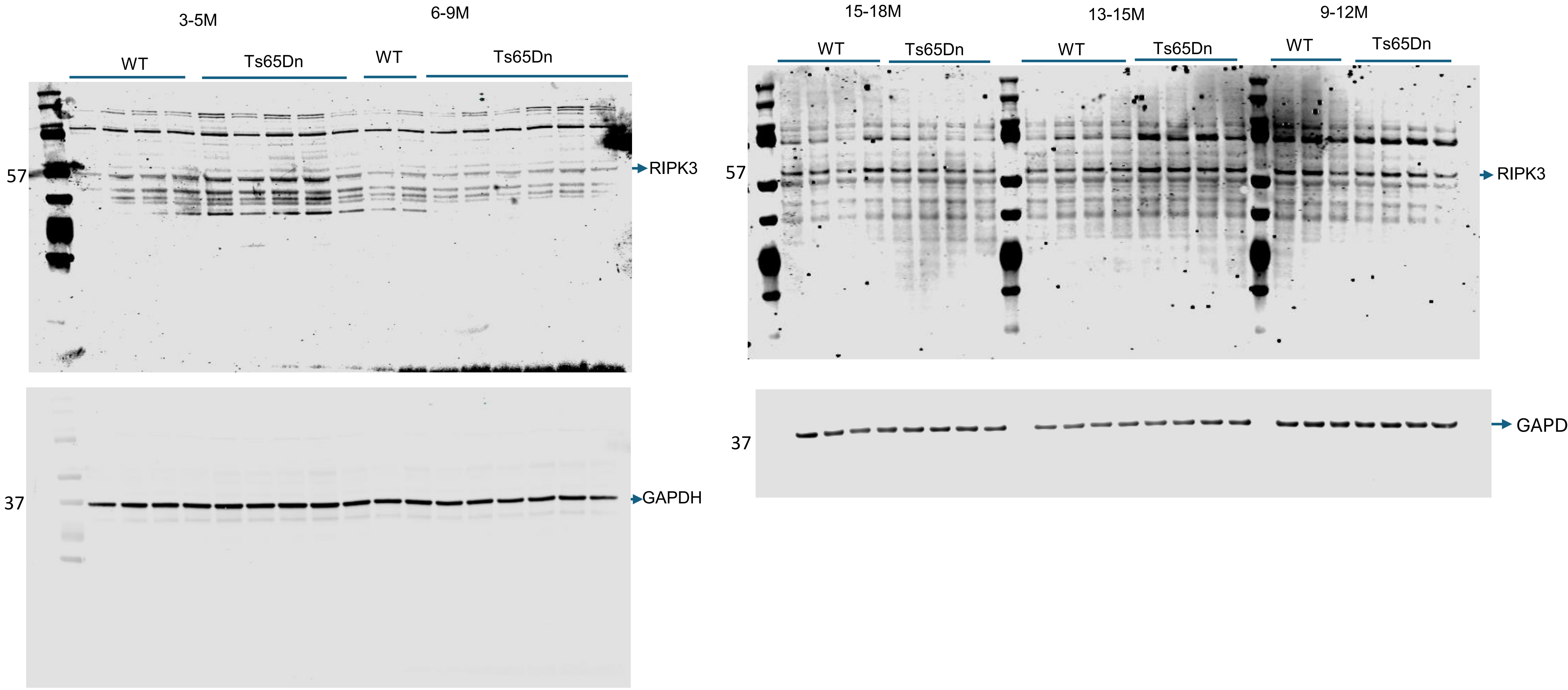
3-5M
6-9M
WT
Ts65Dn
WT
Ts65Dn
57
RIPK3
37
GAPDH
15-18M
13-15M
9-12M
WT
Ts65Dn
WT
Ts65Dn
WT
Ts65Dn
57
RIPK3
37
GAPD

Figure-1(e)

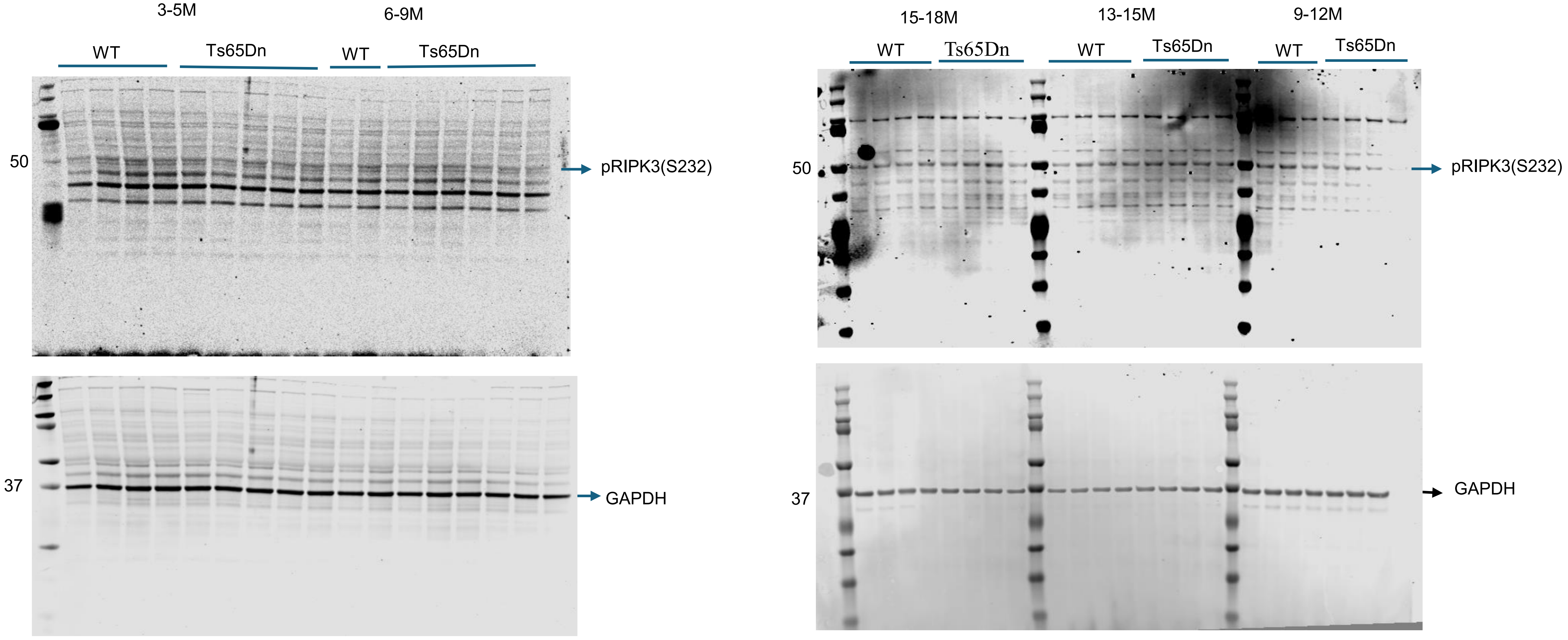

3-5M
6-9M
WT
Ts65Dn
WT
Ts65Dn
50
pRIPK3(S232)
37
GAPDH
15-18M
13-15M
9-12M
WT
Ts65Dn
WT
Ts65Dn
WT
Ts65Dn
50
pRIPK3(S232)
37
GAPDH

Figure-1(g)

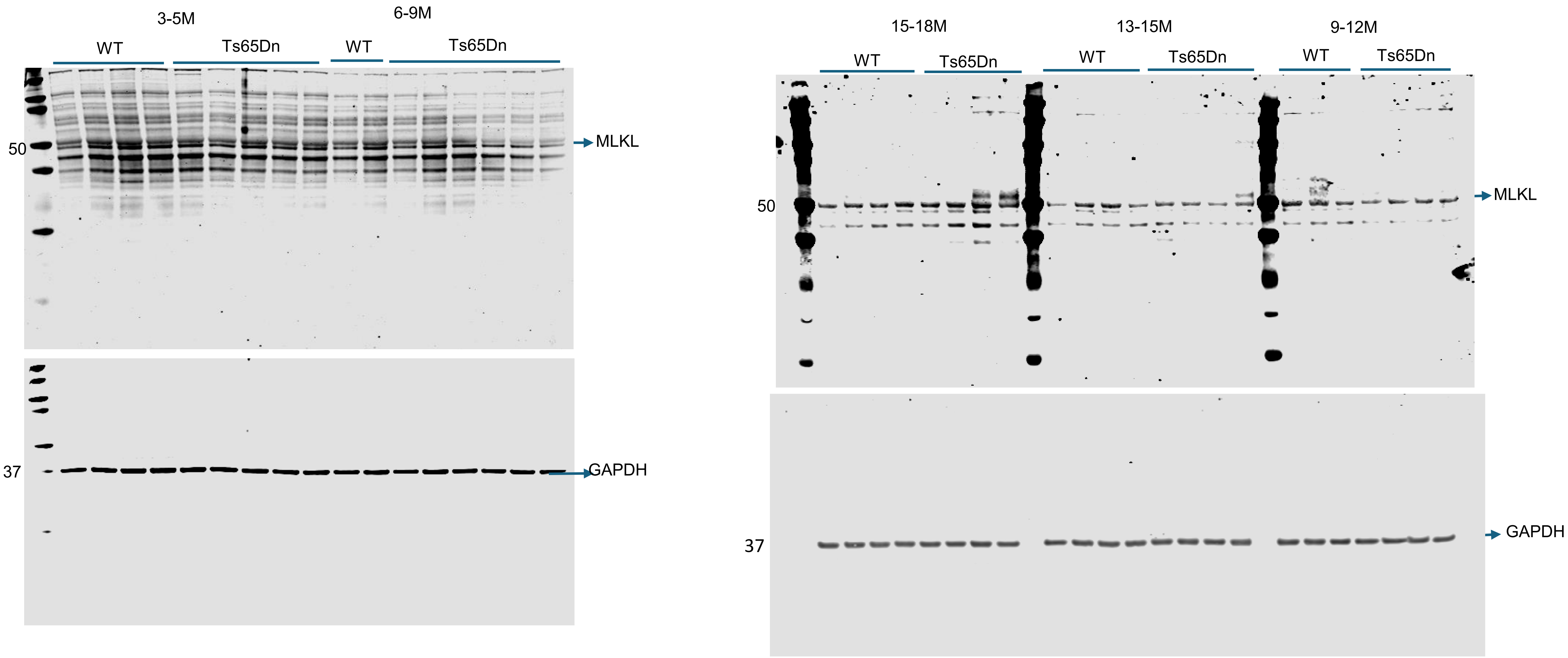
3-5M
6-9M
WT
Ts65Dn
WT
Ts65Dn
50
MLKL
37
GAPDH
15-18M
13-15M
9-12M
WT
Ts65Dn
WT
Ts65Dn
WT
Ts65Dn
50
MLKL
37
GAPDH

Figure-1(h)

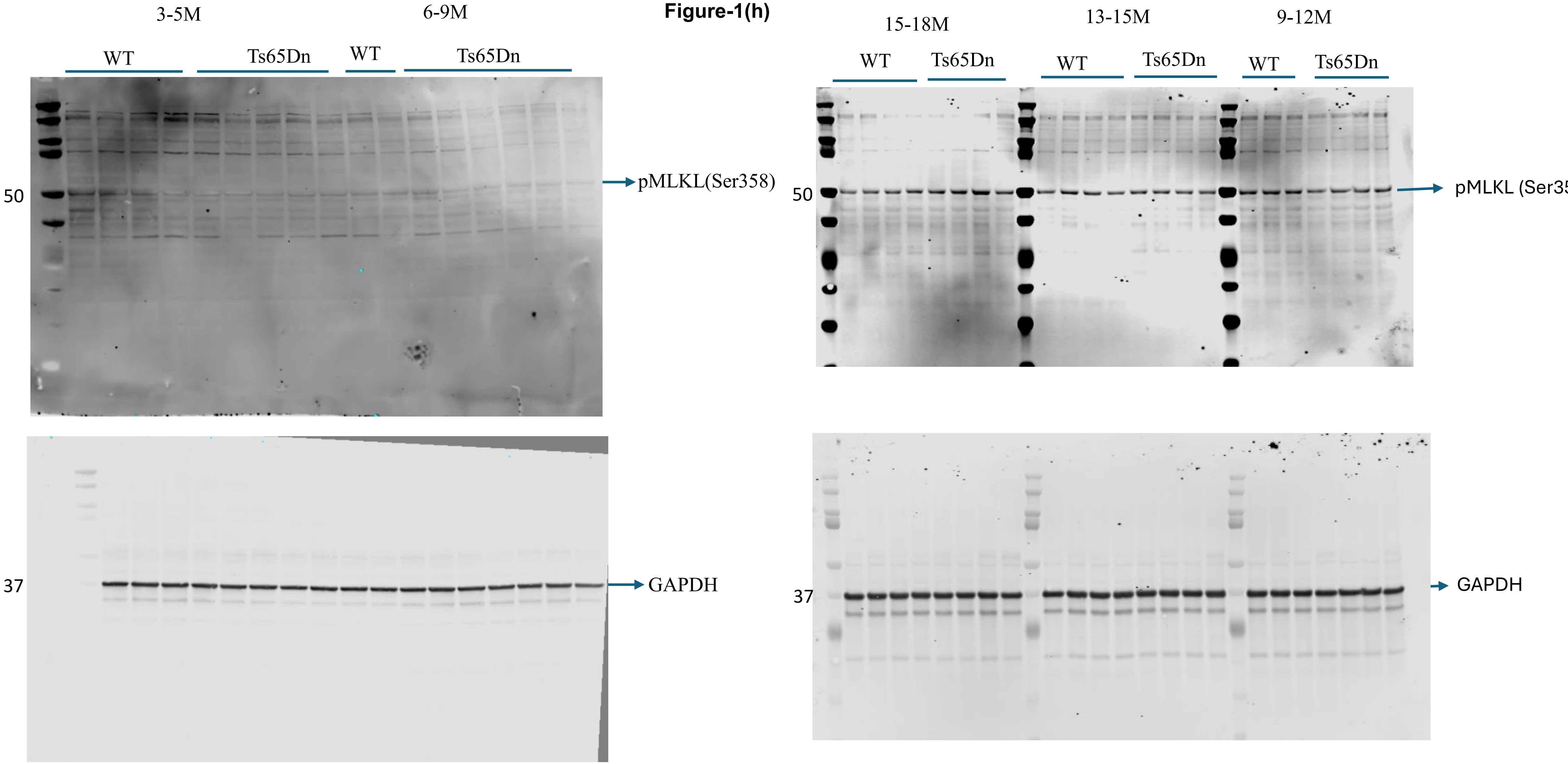
3-5M
6-9M
WT
Ts65Dn
WT
Ts65Dn
50
pMLKL(Ser358)
37
GAPDH
15-18M
13-15M
9-12M
WT
Ts65Dn
WT
Ts65Dn
WT
Ts65Dn
50
pMLKL (Ser35
37
GAPDH
